# The Gemstone Cyborg: How Diamond Films Are Creating New Platforms for Cell Regeneration and Biointerfacing

**DOI:** 10.3390/molecules28041626

**Published:** 2023-02-08

**Authors:** Nádia E. Santos, Joana C. Mendes, Susana Santos Braga

**Affiliations:** 1 LAQV-REQUIMTE, Department of Chemistry, University of Aveiro, 3810-193 Aveiro, Portugal; 2 Instituto de Telecomunicações and University of Aveiro, 3810-193 Aveiro, Portugal

**Keywords:** NCD, UNCD, NSCs, neurons, cells, adhesion, differentiation, biointerfacing

## Abstract

Diamond is a promising material for the biomedical field, mainly due to its set of characteristics such as biocompatibility, strength, and electrical conductivity. Diamond can be synthesised in the laboratory by different methods, is available in the form of plates or films deposited on foreign substrates, and its morphology varies from microcrystalline diamond to ultrananocrystalline diamond. In this review, we summarise some of the most relevant studies regarding the adhesion of cells onto diamond surfaces, the consequent cell growth, and, in some very interesting cases, the differentiation of cells into neurons and oligodendrocytes. We discuss how different morphologies can affect cell adhesion and how surface termination can influence the surface hydrophilicity and consequent attachment of adherent proteins. At the end of the review, we present a brief perspective on how the results from cell adhesion and biocompatibility can make way for the use of diamond as biointerface.

## 1. Introduction

Diamond has a set of properties that make it a promising biomaterial to be used as new platform for cell growth, regeneration, and biointerfacing. The first and foremost factor of biocompatibility of diamond is its chemical inertness, which makes it also bioinert. The biocompatibility of diamond is expressed by various other factors: (i) little or no immunogenic response, that is, no inflammation or response by immune cells is triggered when a diamond film is implanted into the body; (ii) non-thrombogenic, that is, it causes no coagulation because it does not induce platelet aggregation; (iii) non-toxic to the body even when diamond nanoparticles are released [1]. In addition to its biocompatibility, diamond fulfils a set of requirements for growing living cells in the laboratory, such as minimal effect in the normal cell growth cycle, lifetime, and functionality [2]. Another property of diamond that makes it a promising tool for biomedical applications is its electrical conductivity. Native diamond is not conductive, but lab-grown diamond thin films deposited in H-rich environment do possess surface conductivity [3]. This property can be further tailored by adding small amounts of non-carbon elements acting as electron donors or receptors, such as boron, nitrogen, and phosphorus. This process is called doping, with boron having a well-known vital role in creating *p*-type conductivity [4]. In tandem, the hydrophilicity and consequent biocompatibility of diamond can be improved by terminating the surfaces with ether (C–O–C), carbonyl (C=O) and hydroxyl (C–OH) groups [1]. These terminations, as we shall see in an upcoming section, can play an important role in cell culture. Besides simple uniform functionalisation, the surface of diamond films can be easily functionalised and consequently contain specific regions that sustain cell growth and regions that do not. Last but not least, the cost of artificially producing diamond films has become increasingly lower and this material is nowadays far more accessible than gemstone diamond: a plate of diamond with 1 cm^2^ in size and a thickness of 0.5 mm can be produced for approximately 46 euros (50 US dollars). In addition, in some cases, the diamond plate can be replaced by a layer of a different material (such as silicon) covered with a few micron-thick film of diamond, which can be produced at a fraction of the cost [1].

Recently, based on the unique set of characteristics of diamond, researchers have been inspired to explore its biomedical applications, aiming at improving the quality of life of individuals suffering from degenerative diseases or dysfunction of human tissue. This goal can be met by using diamond as a high-performance substrate for the culture of non-differentiated cells, such as neural stem cells (NSCs) [5,6], human-induced pluripotent stem cells (IPS) and IPS-derived neuronal progenitors [7], various types of neurons [8,9,10], cells from the connective tissue such as fibroblasts [11,12,13,14] and osteoblasts [15,16,17], epithelial cells [18], among others [1]; and by using it to create biointerfaces [19]. 

In this review, we will focus on the use of diamond surfaces as platforms for the adhesion and proliferation of cells and describe their impact on cell differentiation. We will take into consideration the morphological characteristics of the films, which depend strongly on the method and conditions used in its deposition. To provide the non-familiarised reader with the required concepts, Section 1.1 will briefly describe the growth of diamond by chemical vapour deposition (CVD) and the impact of the process parameters on the film’s morphology. Grain size, in particular, is an important factor for cell growth, as it may influence the adhesion of cells to the surface and even their development cycle (more details in Section 2). The last section of this review (Section 3) presents some exciting results on the use of diamond as a functional electric platform for biointerfacing, including the development of microelectrode arrays (MEAs) for signal cell reception and retinal implants.

### 1.1. Growth of Diamond by Chemical Vapour Deposition

Diamond is a solid form of pure carbon where the atoms follow a tetrahedral arrangement, each atom binding to the four nearest atoms by means of sp^3^ bonds. These bonds are extremely strong and originate the extreme properties of this material. Natural diamond forms inside the Earth mantle, at a depth of 150 to 200 km, with pressures ranging between 4.5 and 6.5 GPa and temperatures of 900 to 1300 °C. 

**Figure 1:**
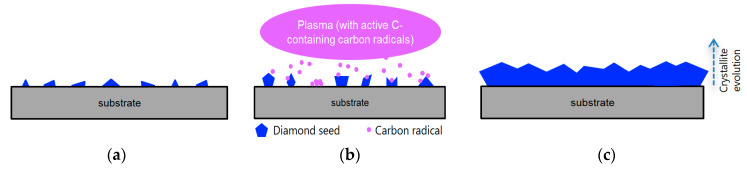
Schematic representation of the various steps of chemical vapour deposition (CVD) diamond growth on non-diamond substrates: (**a**) substrate surface enriched with diamond seeds, (**b**) seeded substrate exposed to diamond growth conditions, and (**c**) coalesced diamond film.

Diamond can, however, also be prepared by artificial methods such as CVD. In this case, growth occurs below atmospheric pressure and involves the deposition of carbon atoms that originate from the dissociation of a carbon-containing gas precursor on a solid substrate. To this end, a mixture of gases containing a carbon source is fed into a chamber and a high energy source such as microwaves or a heated filament creates a plasma by splitting the gas molecules into chemically active radicals. In the case of a non-diamond substrate, an extra seeding step is required since the activated carbon-containing radicals will not nucleate spontaneously on non-diamond materials. This step places diamond nucleation sites on the substrate surface (Figure 1a); these sites, once exposed to the plasma (Figure 1b), grow three dimensionally until they coalesce and form a closed film (Figure 1c). The grain boundaries that are formed between adjacent grains of the diamond film contain non-diamond molecules and materials, such as ordered and disordered graphite, amorphous carbon, and transpolyacetylene. When the individual diamond crystals coalesce into one another, they start growing in the form of columns with an orientation that is roughly perpendicular to the substrate surface. The average grain size gets larger as the film thickness increases [20], with individual grains having a randomly oriented texture that translates into a high surface roughness. 

Different carbon-containing molecules can be used as the precursor, methane being the most common. The films are typically deposited at temperatures in the 700–800 °C range and in hydrogen-rich environments (1–2% of methane diluted in hydrogen), which causes the surface to be terminated with hydrogen. The thickness and the grain size of the deposited films can vary between a few and hundreds of micrometres; therefore, they are usually named micro or polycrystalline diamond films. Grain size can be reduced by properly tuning the composition of the starting mixture of gases and the surface temperature to promote re-nucleation of the diamond crystals [21]. In this case, the grain size of the deposited films may range from a few nanometres up to a hundred nanometres (increasing with film thickness, because of the columnar growth) and the deposited films are commonly referred to as nanocrystalline diamond (NCD). NCD films are also deposited in hydrogen-rich CVD environments but, because of their smaller crystallite size, the density of grain boundaries *per* surface area is higher than in the case of microcrystalline diamond films. 

Finally, ultrananocrystalline diamond films (UNCD) are deposited in argon-rich (up to 99% in composition), hydrogen-poor CVD environments. UNCD has a typical grain size of 2–10 nm [22] independently of the film thickness. This happens because, unlike microcrystalline diamond and NCD that have a columnar structure, the grains in UNCD are embedded into a non-diamond matrix. UNCD films have, thus, an even higher density of grain boundaries than NCD films. 

**Figure 2:**
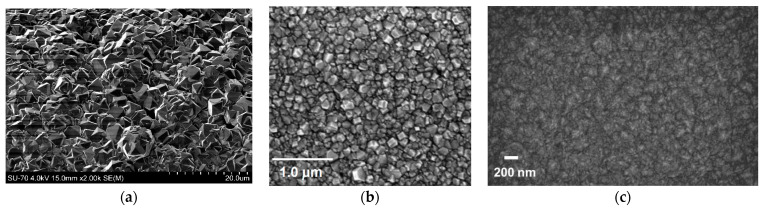
SEM micrographs showing the morphology of: (**a**) microcrystalline diamond; (**b**) nanocrystalline diamond (NCD); (**c**) ultrananocrystalline diamond (UNCD). (**b**) is reprinted with permission of Elsevier from Diamond and Related Materials, Vol. 15, O.A. Williams, M. Daenen, J. D’Haen, K. Haenen, J. Maes, V. Moshchalkov, M. Nesládek, D.J. Gruen, Comparison of the growth and properties of ultrananocrystalline diamond and nanocrystalline diamond, pp. 654–658 [23], ©Elsevier, 2006. (**c**) is reprinted with permission from Sumant, A.; Grierson, D.; Gerbi, J.; Carlisle, J.; Auciello, O.; Carpick, R., Physical Review B, 76, 235429, 2007, © American Physical Society, 2007 [24].

Scanning electron microscope (SEM) images depicting the typical surface of microcrystalline diamond, NCD, and UNCD are shown in Figure 2. 

It is worth noting that both microcrystalline (Figure 2a) and NCD films (Figure 2b) present a faceted structure, with the individual crystals clearly distinguishable, and a randomly oriented texture. Films composed of UNCD (Figure 2c), in turn, are characterised by lacking a proper microstructure. Most importantly, UNCD films have a high volume fraction of grain boundaries. 

## 2. Diamond as a Substrate for Cell Culture

Cell culture is an important tool not only for observing and studying cells, but also for growing new cells and even tissue that could, ultimately, be employed in the treatment of cellular degenerative diseases, *e.g.*, central nervous system ailments caused by loss or damage of a specific sub-population of the brain cell lineages [25,26]. This section presents details on the use of diamond substrates as biocompatible platforms for cell adhesion and proliferation and, in some cases, for the promotion of cell differentiation. 

### 2.1. Fibroblasts 

Fibroblasts are ubiquitous cells in the human body, responsible for creating connective tissue and for the synthesis of collagen, glycogen, and other components of the extracellular matrix (ECM). They are specialised in keeping the various cells that form our organism together. Fibroblasts can easily adhere to a substrate because they contain the adequate biochemical machinery for the formation of their own ECM. 

In general, cell adhesion to the ECM is a sequential process in which different stages can be identified over time. Detached cells, such as the ones that are being cultured from a suspension, start by attaching to the substrate. In the second stage, attached cells undergo spreading, which results in flattening. Subsequently, some cell types, such as, for instance, fibroblasts, epithelial cells, and immune cells, can undergo migration through the ECM, whereas others remain stationary. Each one of these stages of adhesion, *i.e.*, attachment, spreading, migration/immobilisation, involves changes in morphology and cytoskeletal structure [27].

**Scheme 1:**
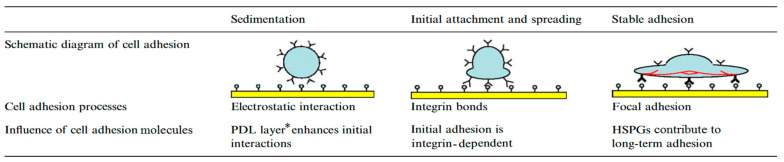
Stages of *in vitro* mechanical cell adhesion, studied using epithelial cells and showing the main physical and biomolecular processes involved: electrostatic interaction in the sedimentation stage, integrin-mediation in the attachment/spreading stage, and production of heparan sulphate proteoglycane in the stage of stabilisation of the adhesion. * PDL = poly-D-lysine, an adhesion-promoting molecule, was added to the substrate; HSPG = heparan sulphate proteoglycane. Adapted with permission from Hong *et al.* [28].

An example of the different stages involved in cell adhesion is given in Scheme 1. The study monitored adhesion of endothelial cells from bovina aorta to a solid surface and evaluated the influence of biomolecules and biophysical factors on the adhesion process. Laminin was shown to accelerate initial adhesion by sedimentation, which was explained by the augmented superficial electrical charge, while the spreading stage involved the production of integrin. [28]. 

#### 2.1.1. Growth of Fibroblasts on UNCD Substrates

Shi *et al*. studied the culture of mouse embryonic fibroblasts (harvested at day 18) on a collection of UNCD substrates, testing a combination of parameters such as absence of doping, doping with nitrogen, and H- surface termination (*versus* no surface treatment) [11]. All UNCD substrates were shown to support fibroblast adhesion and consequent proliferation, albeit with lower cell densities than those observed for the control, a commercial cell culture dish. The authors ascribed adhesion in UNCD to its nanometric structure: the films had a small grain size of 2–5 nm, and a correspondingly high density of grain boundaries. The exposure of non-doped UNCD films to H plasma decreased the adhesion of the cells, when compared to the untreated non-doped UNCD surfaces; in contrast, the N-doped UNCD film treated with H showed the highest adhesion, due to the possible formation of N–H and NH_2_ bonds on the surface of the films. 

Chong *et al*. corroborated that UNCD is, *per se*, capable of promoting fibroblast adhesion and growth. Using films with grain sizes within 5–10 nm and normal human fibroblasts, good adhesion and proliferation was observed, which was, once more, attributed to the fine-grain and small surface corrugation that afford a wide surface contact area with the cells [12]. Notably, surface functionalisation with hydroxyl or carbonyl groups did not result in any significant increase in cell density, which indicates that the morphology of UNCD provides sufficient conditions for fibroblast culture. 

Tong *et al*. reported the growth of mouse embryo fibroblasts (3T3 cell line) on UNCD films with slightly larger grain size, lying within the 5–30 nm interval [13]. Films were treated with different methods for surface hydrophilisation and sterilisation, with exposure to hydrogen peroxide aerosol affording the best outcome: cell density after two days of incubation was significantly higher than that of the control (a glass plate). Fibroblast growth on the other diamond samples was also observed, but with lower cell density values than the control.

#### 2.1.2. Growth of Fibroblasts on Large-Grain NCD Substrates

Chong *et al*. prepared NCD films with 100–300 nm grain size and treated their surface by ultraviolet (UV) oxygenation and UV photochemical grafting with undecylenic acid to test the effect of these groups contribution to make larger grained films adequate for growing fibroblasts [12]. Samples with the photochemically grafted acid and UV oxygenation treatments presented increased values of cell density, by about 5- and 6-fold, respectively, when compared to non-functionalised substrates. This way, introducing carbonyl and hydroxyl groups onto the surface of diamond films seems to be a good approach for increasing cell adhesion, mainly by providing tethering sites for laminin. This cross-linked polypeptide plays an important role in cell adhesion by interacting with cell-surface receptors and several ECM adhesive molecules (other laminin molecules, collagen, thrombospondin, elastin, fibronectin, and vitronectin) to stabilise the ECM as a whole structure [29]. 

### 2.2. Kidney Epithelial Cells

Epithelial cells are found not only in skin but also in the lining of a variety of organs, from blood vessels to airways and the digestive and urinary tracts. Growing these cells *in vitro* is an important goal in tissue engineering. 

An approach to the culture of this type of cells was reported by Lechleitner *et al*. using MK-2 cells, an immortalised cell line derived from human renal epithelial proximal tubular epithelium [18], which is easier to manipulate than primary cells. The researchers used substrates of H- and O-terminated NCD films, prepared by hot filament chemical vapour deposition (HFCVD), having a mean grain size of 70 nm and roughness within 10–12 nm; non-treated borosilicate glass was used as the control. The results demonstrated that H-NCD inhibited cell attachment while, in turn, cells were able to attach to the hydrophilic O-NCD substrates. The mechanisms of adhesion were also investigated. Knowing that the interaction between cells and the surface depends on the integrin–ligand complex formation that initiates intracellular signalling cascades to start the attachment process, researchers quantified one of the main signalling molecules, focal adhesion kinase (FAK). As expected, higher quantities of FAK were identified in cells growing on O-NCD. Flow cytometry analysis showed that the percentage of cells in the S phase, that is, the cell cycle phase in which DNA synthesis occurs, was significantly increased on O-NCD as compared to all other surfaces, thus indicating that this substrate has the ability to induce proliferation.

### 2.3. Osteoblasts and Osteoblast-like Cells

NCD is an excellent platform for growing bone tissue because it can stimulate adhesion, proliferation and even differentiation of osteoblasts [15,17], the cells responsible for the de novo synthesis of bone mineral in a living organism. Growing these cells *in vitro* is thus a helpful strategy to generate biomaterial applicable in the treatment of various orthopaedic ailments.

#### NCD as Coating for Prosthetic Implants

NCD films are hard (their hardness approaches the hardness of bulk diamond) [30], present a suitable fracture toughness, and are resistant to wear. Moreover, their roughness mimics the surface of the bone and they are biocompatible, which makes them candidates to be used as coating layer of prosthetic implants. Moreover, NCD films possess high chemical resistance and are stable in the presence of a leaching agents, thus being a very safe material for implants, especially when compared to bone cement and chromium–cobalt particles [31]. The biocompatibility and lack of toxicity of NCD and NCD/amorphous carbon composite films was demonstrated on human osteoblast-like SAOS-2 cells [32,33]. 

Amaral *et al*. reported the growth of immortalised osteoblast-like cells (MG63 line) and human bone marrow cells on NCD substrates [17]. The films were deposited on Si_3_N_4_ surfaces by HFCVD with an Ar–CH_4_–H_2_ gas mixture. Being a highly proliferative line, MG63 osteoblast-like cells were well attached and spread after only one day of culture and completely covered the material surface after three days of culture (growth rate was similar to that of the control substrate, a standard polystyrene plate) [17]. In comparison, human primary bone marrow cells required a whole week to fully complete spread and cover the whole NCD surface. Cell growth was accompanied by the production of a fibrillar matrix, observed at day 14, and by mineral deposition, observed at day 21. Similar growth occurred on the control polystyrene substrate, although with lower production of fibrillar matrix and lower mineral deposition. Noteworthy, from day 7 onwards, cell viability measured by the MTT assay was significantly higher on the NCD films. The total protein content and alkaline phosphatase (ALP) activity were also measured, both being significantly higher after day 7 on NCD films regarding the control. These results show that, aside from inducing human osteoblast proliferation, NCD also stimulated cell metabolic activity. 

**Figure 3:**
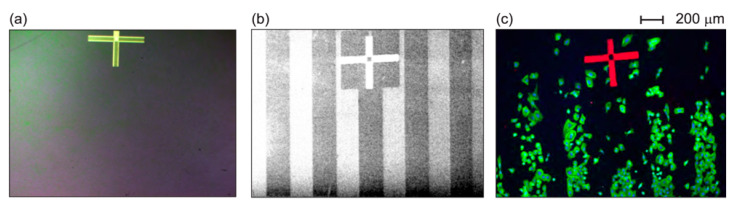

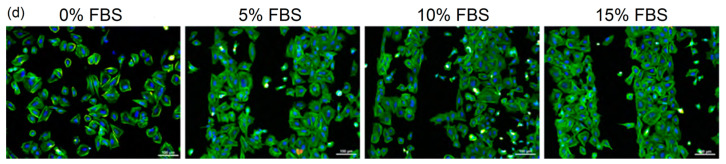
NCD film with 200 μm wide H/O-terminated patterns: (**a**) optical (bright field) image prior to cell plating showing optically transparent and featureless surface, (**b**) SEM image prior to cell plating where bright stripes correspond to H-termination and dark stripes to O-termination of the diamond surface due to their opposite electron affinity. Fluorescent microscopy images of osteoblastic cells (SAOS-2) cultivated on the substrate, showing (**c**) the alignment cross for correlation of the surface termination micro-patterns with the cells, and (**d**) cell growth, after 2 days of incubation, on 200 μm H/O-terminated stripes on diamond films in McCoy’s medium supplemented with different foetal bovine serum (FBS) concentrations (0, 5, 10, and 15%). Reproduced from Rezek *et al*. [15] under a creative commons licence.

Rezek *et al*. reported the adhesion of osteoblast-like SAOS-2 cells, an immortalised cell line which keeps constant properties for long periods of time, onto diamond surfaces [15]. In this study, the researchers deposited high quality NCD films with crystallite size around 50 nm on both sides of silicon substrates by microwave plasma chemical vapour deposition (MPCVD). To investigate the possibility of shaping cell growth into a pattern, the NCD films were lithographically processed to generate alternating H- and O-terminated patterns of 30 to 200 µm width. The contribution of other factors to cell patterning was also tested, namely the cell density at the seeding phase, at 2500 *vs*. 10,000 cells/cm^2^, and the presence or absence of foetal bovine serum (FBS). After 48 h of incubation, fluorescence microscopy showed that preferential cell adhesion onto the O-terminated stripes occurred on the plates having a low initial cell density (2500 cells/cm^2^) and cultivated in the presence of FBS (Figure 3), which was a clear indication that the proteins contained in this serum were guiding cell adhesion. When an initial density of 10,000 cells/cm^2^ was used, the cells did not show a selective growth on certain spots, having colonised the hydrophobic stripes as well as the hydrophilic ones. The same occurred when no FBS was present in the culture medium. 

### 2.4. Neural Stem Cells and Neurons

#### 2.4.1. Stem Cells Differentiation with UNCD Films

The adhesion and growth of NSCs to UNCD was investigated by Chen *et al*. [5]. Two different diamond substrates were tested, both made of UNCD with a roughness within the nanometre range (deposited by MPCVD on quartz dishes) and having different post-treatments: one was finished in a plasma of pure hydrogen in order to obtain a H-terminated surface (H-UNCD), and the other was exposed to oxygen plasma for an O-terminated surface (O-UNCD). The NSCs were isolated from mouse embryos at day 11.5 according to a previously described method [34] and cultured for 7 days before the cell proliferation assay, which consisted in placing NSCs, with a seeding density of 1.5 × 10^4^ cells/cm^2^, in sample-holding cells on the surface of both H- and O-UNCD, and in polystyrene Petri dishes, for comparison. Cell cultures were allowed to grow for 4 and 7 days and subsequently stained with trypan and harvested for counting on a haematocytometer. Primary NSCs had a good proliferation on both H- and O-terminated diamond surfaces, with a slightly better result for H-UNCD. In addition, real-time polymerase chain reaction (RT-PCR) of cells grown on the two kinds of diamond surfaces, using two cell differentiation markers, showed low expression of the SOX2 gene, a transcription factor essential for maintaining cell pluripotency, and increased expression of SOX1, a transcription factor that functions primarily in neurogenesis. Immunostaining showed that H-UNCD favoured neuron differentiation, because the cells cultured on it had increased levels of βIII tubulin (in comparison to the polystyrene control), even when they were not supplemented with differentiation factors. In turn, O-UNCD favoured differentiation into oligodendrocytes (measured by increased expression of glial fibrillary acidic protein). These results are quite unexpected because differentiation into neurons is reported to occur preferentially in soft matrices that mimic the density of brain tissue [30]. Another quite interesting aspect about these results is that they open way for the application of UNCD to promote the differentiation of NSCs into different lineages just by changing the diamond’s surface. Taking this into account, Chen *et al.* further evaluated neurosphere formation on the UNCD substrates starting from the NSCs; after 10 days of incubation, the efficiency of neurosphere formation was 0.43%. The neurosphere assay is a well-known tool for evaluating the behaviour of NSCs, not only in embryos but also in adults [35], thus validating the use of UNCD as a platform for brain cell differentiation. It is important to highlight, however, that neurosphere formation was only observed on H-UNCD or O-UNCD films without any adherent cells, indicating that UNCD film is a more favourable culture surface for use in neural differentiation rather than stem cell growth. 

**Figure 4:**
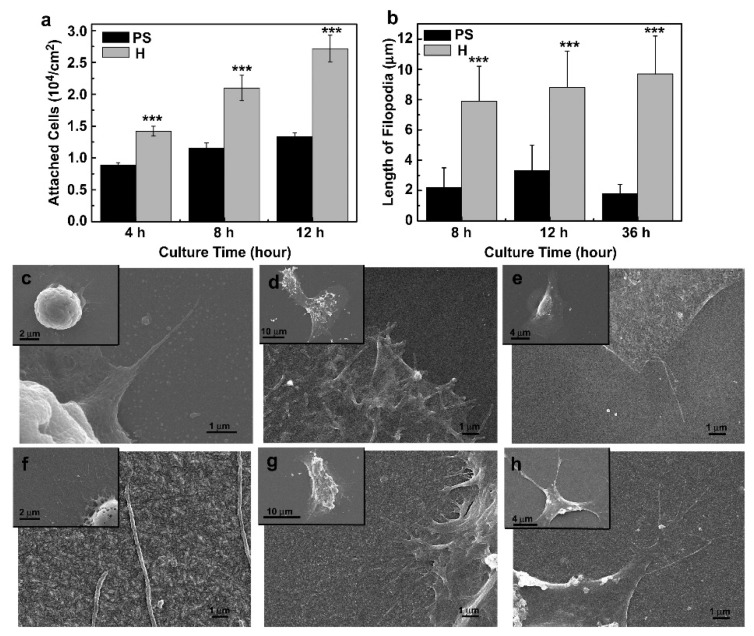
Comparison of: (**a**) adhesion of neural stem cells (NSCs) (from a seeding density of 3.5 × 10^4^ cells/cm^2^) to a polystyrene (PS) Petri dish (black bars) and to H-UNCD films (grey bars), with data expressed as mean ± standard deviation (*n* = 4); (**b**) length of filopodia (100 nm in width) of NSCs cultured on polystyrene (black bars) and H-UNCD films (grey bars), in supplemented medium, at 8, 12, and 36 h of culture. More than 30 cells were randomly picked and measured for each sample by an unbiased investigator who was not informed of the nature of each sample. SEM micrographs, at low (inset) and high magnification of NSCs cultured for 8, 12, and 36 h, on: (**c**–**e**) polystyrene, showing short filopodia with few protrusions that do not spread far from the main cell body; (**f**–**h**) H-UNCD, showing numerous, long and complex filopodia that extend away from the main cell body. Significance levels: *** *p* < 0.001 [6].

In a follow-up study [6], the same research group focused on the H-UNCD films, corroborating their ability to induce neuronal differentiation on NSCs in a spontaneous manner, that is, without the use of any differentiation factors, and showing the morphological advantages for cell adhesion of the surface of H-UNCD in regard to a smooth material like polystyrene: H-UNCD films promoted better NSC adhesion (by roughly twice as much as in polystyrene) and allowed for a more extensive spreading of the cells, with the development of more filopodia and complex protrusions that extended far away from cells, possibly indicating more binding sites for focal contact formation between cells and H-UNCD films (Figure 4). 

**Scheme 2:**
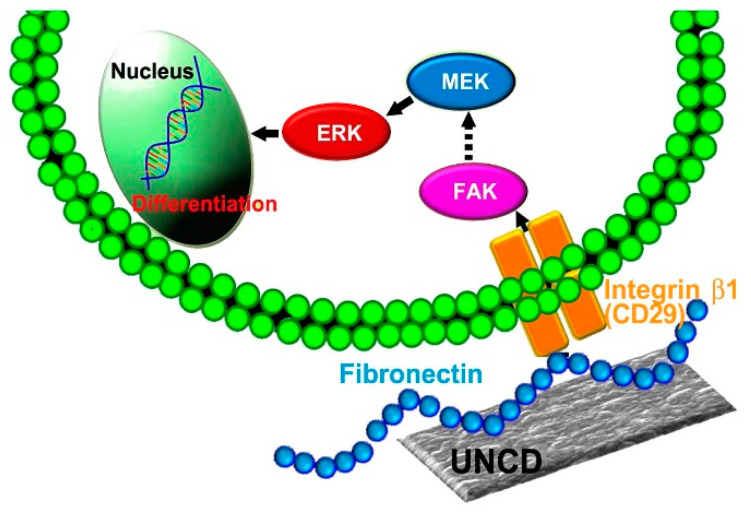
Schematic representation of the role of H-UNCD films in mediating differentiation from NSCs. Adsorbed fibronectin on H-UNCD surface activates integrin, Fak, and Erk1/2 pathways which trigger NSC differentiation into neurons [6].

Collection and analysis of proteins from the culture medium showed that the higher cell adhesion in H-UNCD films was associated with higher expression of fibronectin, a protein involved in cell adhesion as well as in cell differentiation. The presence of high fibronectin levels was shown to upregulate the expression of integrin β1, and to ultimately lead to the activation of the mitogen-activated protein kinase/extracellular signalling-regulated kinase1/2 (MAPK/Erk1/2) and to the differentiation of NSCs into neurons (Scheme 2).

Considering that the ECM environment of the central neural system plays an important role in the differentiation of NSCs during both birth and adulthood, these results are quite promising because they show that a selective modification of the surface terminal groups in UNCD permits mimicking ECM conditions in the laboratory and offers the possibility of bioengineering, in a laboratory setting, the production of neurons and/or oligodendrocytes that can later be used for transplantation and tissue engineering. 

#### 2.4.2. Culture of Differentiated Neural Cells on Diamond Surfaces

The previous subsection described how UNCD favours the differentiation of NSCs instead of their proliferation. As the neural cells become more differentiated, they will also adhere and grow on diamond substrates, from single-crystal diamond [9] to synthetic diamond films grown by CVD [8,10], and even to surfaces that are coated with a layer of nanodiamond seeds [36]. The requirements of cell culture will be, however, more demanding, and strongly dictated by the biology of the cells, with fundamental differences between immortalised neurons and primary neural cultures.

**Immortalised neurons**. The culture of mature neurons is extremely challenging because these cells do not replicate, i.e., once they mature they stop the cell division process [37]. To work around this, one can employ secondary cell lines derived from tumours that have, thus, become immortalised. While differing in physiology from normal neurons, these cell lines have the advantage of being easily grown in cell culture, giving unlimited numbers of cells with minimal variability between cultures. 

P. Ariano *et al*. used immortalised hypothalamus neuron-like cells (GT1-7 line) to investigate the role of diamond surface morphology and atomic termination in cell survival and viability [10]. Two quite distinct morphologies were tested: (i) large-grained (10–400 nm), high-roughness (≈15 nm) NCD films, grown by modified HFCVD on quartz dishes, *vs*. (ii) striated, low-roughness (≈1 nm) diamond films, grown by MPCVD on high pressure high temperature (HPHT) single crystal diamond substrates and having either H- or O- terminated surfaces. Results demonstrated that, while cells were able to adhere to and survive on all the films, cell density at 24 and 48 h on NCD and striated films was significantly different, being *c.a*. 20% higher for those on NCD. Moreover, the films with a striated morphology presented a non-negligible amount of dead and shrunken cells. Other reports had already shown the impairment of cell adhesion in this type of diamond films, because of their low roughness [9,38]. In NCD films, no significant differences regarding surface termination were observed, which was attributed to the fact that the roughness of these films was sufficient for the cells to adhere. 

**Primary co-cultures of neurons and glial cells**. Immature primary neural cells offer a suitable alternative to immortalised ones, while avoiding the problems of non-replicating mature neurons. Primary neural cells are derived directly from live embryonic tissue and dissociated using enzymatic and/or mechanical methods. This means that the cell lysate contains not only neurons but other brain cells such as glia. The culture of primary cells is more challenging than that of immortalised cells, requiring the addition of poly-D-lysine, laminin, or other adhesion factors that promote adhesion through the presence of multiple negative charges. In addition, the use of specific trophic factors is required to promote adequate proliferation and maturation [39]. 

May *et al*. studied how a co-culture of primary cortical neurons and glial cells (isolated from the cerebral cortex of 18-day old embryonic Sprague Dawley rats) can grow on microcrystalline diamond films deposited by HFCVD on silica substrates [8]. Some of the films were doped with boron to investigate a possible role of this element in cell proliferation, boron being a potentially poisonous element for many cells. No significant changes in cell proliferation were observed in comparison with non-doped films, indicating good compatibility of the boron-doped films. Regarding their surface functionality, O-terminated films allowed for cells to proliferate more extensively than the H-terminated ones, but in both cases an adhesion-promoting layer of polylysine was required (otherwise, cells died within a few days). The work of May *et al*. suffers, however, from a relevant flaw: the boron doping rate was not disclosed. A boron doping ratio of 2000 ppm was later reported to afford biocompatible diamond films for neural culture [40].

**Figure 5:**
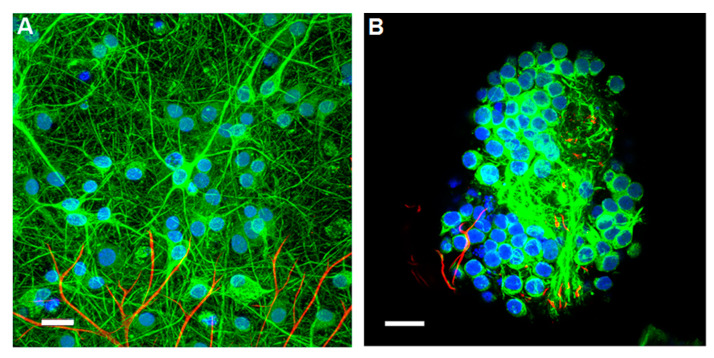
Adhesion and growth of 17-day rat embryo hippocampal neurons and glial cells on (**A**) NCD substrates coated with an adhesion-promoting layer of poly-D-lysine and laminin, and (**B**) virgin NCD substrates, as shown by confocal microscopy images with immunostaining. Cells were stained with anti-neurofilament antibodies (NF, giving neurons a fluorescent green colour), 4′,6-diamidino-2-phenylindole (DAPI, marking the DNA-rich nucleus of neurons with blue) and antibodies for glial fibrillary acidic protein (anti-GFAP, making glial cells appear in red). In each image, scale bar = 20 μm. Adapted from Ojovan *et al*. [41] under a creative commons licence.

Ojovan *et al*. studied the growth of rat hippocampal neurons and glia (isolated from 17-day rat embryos and from 1-day old neonates) on NCD films prepared by MPCVD and presenting crystallites of around 15–17 nm diameter; the films were coated with poly-D-lysine and laminin to promote cell adhesion [41]. The main goal of the study was to demonstrate the relevance of the adhesive coating over film morphology or surface terminal groups (-H or -O). This was confirmed by the results of non-coated diamond films, both H-terminated and O-terminated: in these films, cells tended to aggregate in clusters instead of spreading in monolayers over the substrate (Figure 5). In contrast, the films coated with poly-D-lysine and laminin offered an adhesive substrate for primary neurons, which adhered well and formed an extended, dense network of neurites. 

**Figure 6:**
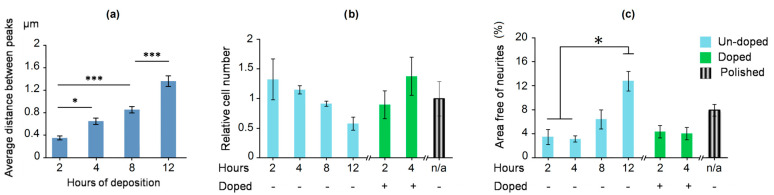
Neural growth in function of diamond surface morphology: (**a**) average distance between sharp peaks on two adjacent crystallites; values represent the average of 20 distances on 4 microscopic fields from 2 samples. (**b**) Number of cell nuclei present on each type of substrate, compared in function of the number of cells growing on the control substrate (polished non-doped diamond) that has been normalised to 1. (**c**) Percentage of area (mean ± s.d., *n* = 3) devoid of neurites for each type of substrate. * *p* < 0.05, *** *p* < 0.001. Adapted from Nistor *et al*. [40] under a Creative Commons licence.

Nistor *et al*. generated a complex, brain-mimicking culture of cells starting from pluripotent NSCs [40]. In the initial steps of differentiation, the stem cells were incubated in polystyrene tissue-culture dishes and matured using differentiation-inducing culture medium; after maturation started (around 50–70 days of culture), they were transferred to polycrystalline diamond substrates prepared by HFCVD. Exposure times used in the preparation of the diamond films ranged from 2 to 12 h. As the time of deposition of the films increased, they presented increasingly larger crystallites and inter-crystallite distances (Figure 6a). While all the diamond films were able to support the long-term survival of the neural cells, cell density differed considerably for films grown with different times of deposition (Figure 6b). The films grown for 12 h, having a distance between peaks (the top of each surface crystallite) above 1 µm, presented many “empty areas”, that is, spots on the diamond surface without any cells. Noteworthy, the number of empty areas in the film grown for 12 h was significantly higher than those observed in the films grown for 2 and 4 h (Figure 6c). This led the authors to conclude that “the efficiency of neurite formation is significantly reduced once the average distance between most conspicuous surface features exceeds 1 μm”. 

Primary co-cultures of hippocampal neurons and glial cells (isolated from 18-day embryos of murines) were incubated for seven days on surfaces coated with a layer of nanodiamond seeds; adhesion was promoted using a mixture of laminin and poly-ornithine (40:60) [36]. Two different types of diamond nano-seeds were used, detonation nanodiamond (DND) particles 6–10 nm in size, and HPHT seeds 20 nm in size. Seeds were dispersed onto the substrates with a variety of different methods, including spin-coating, dip-coating, ultrasonic dispersion, and electrostatic coating using a charged polymer. The layers presented roughness values between 3 and 9 nm and average particle sizes between around 22 nm (for ultrasonic-dispersed DND seeds) and 33 nm (for the spin-coated HPHT seeds). The resulting morphology was correlated with neural growth to show that coatings with smaller average particle size afforded better adhesion and more extended growth of neuron cellular structures (called neurites); nevertheless, it is important to mention that neural cells did grow on all tested coatings and after the seven days of culture they had all formed extended networks over the surface. 

## 3. Diamond as a Biomedical Tool for Biointerfacing

As previously described in Section 1, diamond offers a particular set of characteristics which makes it an excellent tool for the biomedical use. Diamond has been used for other biomedical applications such as biosensors [4,7,42,43] and biointerfaces, also because of its mechanical strength and wear resistance. 

The inertness, biocompatibility, and biosafety of diamond are the first requisites for it to be used in biointerfacing. These properties have been thoroughly described and demonstrated in Section 2 of the present work. The second step is to demonstrate its ability to receive cell communication—in layman’s terms, to “listen” to the cells—and, thirdly, to use it to actively convey information, *i.e.*, to “talk” to the cells. 

### 3.1. “Hello, Is There Anybody out There?”—Cell Signal Reception with Diamond-Based Devices

**Figure 7:**
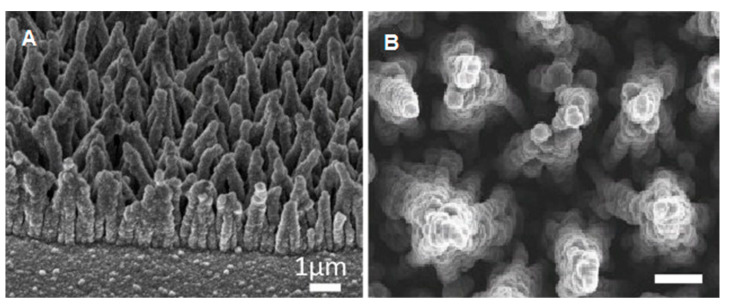
SEM image of the 3D-nanostructured BDD MEA, (**A**) viewed from the side to show the interface of the BDD and the 3D-nanostructured BDD, and (**B**) viewed from the top to show the topography (Scale bar = 600 nm). Adapted from Piret *et al*. [46] under a Creative Commons licence.

MEAs fabricated with boron-doped NCD were shown to successfully record spontaneous electrical activity in rat primary cortical neuronal cultures, without interfering with the passive properties of the cell membrane nor in the active firing response [44]. Biocompatibility and biointegration of boron-doped NCD MEAs were evaluated *in vivo* by implanting them into rat brain, with some subcutaneous inflammatory reaction due to surgery still being visible after two months on MRI scans of the rat brain, but not observed in a second set of scans conducted six months after surgery [45]. Increased signal reception with boron-doped NCD MEAs can be achieved by shaping them into a tri-dimensional nano-structured pattern: Piret *et al*. coated carbon nanotubes previously grown on boron-doped diamond (BDD) with a 50 nm layer of BDD, thus generating an intricate and porous 3D nanostructured MEA surface (Figure 7) [46]. The nano-3D MEAs were shown to detect both electrical burst of electrical signal spikes and small amplitude spiking signals (within the 10–20 μV range) in *ex vivo* mouse embryonic hindbrain-spinal cord. 

The impact of the height of the carbon nanotubes on the growth and attachment of primary cortical neurons (harvested from 18-day rat embryos) to the 3D patterned substrates was evaluated in another study [47]. Arrays of hydrophilic carbon nanotubes with 1, 2, and 3 μm in height were encapsulated by 20 nm-thick BDD films and coated with poly-L-lysine for cell attachment. Only 5% to 25% of the cell membrane was attached to the surface of the 3D substrates. Unexpectedly, a similar percentage (14% to 24%) was quantified for cells attached to flat control samples, suggesting that only around 5% to 25% of cell attachment is needed for proper cell attachment and growth.

### 3.2. Diamond-Based Retinal Prostesis for Artificial Vision 

A pioneer field of diamond application in biointerfacing is the development of retinal implants. 

Hadjinicolaou *et al*. [48] fabricated nitrogen-doped UNCD electrodes and used them to electrically stimulate excised wholemount rat retina. These results suggested that nitrogen-doped UNCD is an electrochemically viable material for retinal stimulation. The compatibility of NCD with retinal cell cultures *in vitro* has also been demonstrated [49].

Rousseau *et al*. [50] fabricated three-dimensional boron-doped NCD soft implants on polyimide with the electrodes located at the bottom of a cavity and a common return ground electrode placed above at the surface of the implant. The soft implants were placed in the sub-retinal region of P23H rats, which are a clinical model for *retinitis pigmentosa* (an untreatable, inherited degenerative photoreceptor disease), over a period of 11 weeks. Subsequently, the retina was explanted together with the implant for histological studies. Confocal microscopy showed that the retina adopted the shape of the implant, while cell labelling revealed the cavities of the implant had been colonised by a small group of bipolar cells (the neurons that make the interface between retinal cells and the central nervous system). These electrodes exhibited both an improvement of the spatial resolution of the stimulation and a long-term bio-inertness. 

In a different approach, Ganesan *et al*. proposed a method for fabricating an all-diamond, hermetic electrical feedthrough array consisting of conducting nitrogen-doped channels within an insulating polycrystalline diamond substrate for a retinal prosthesis [51]. The team later developed a process for integrating nitrogen-doped UNCD electrodes into a custom-built application specific integrated circuit (ASIC) [52] and a prototype hermetic diamond-coated array that used a total of 256 electrodes [53] was finally fabricated. This design allowed the generation of patterns using multiple electrode stimulation and adequate electrical pulses. When implanted on rat retina and evaluated for histocompatibility, no permanent signs of cell toxicity or damage beyond those of the surgical intervention were observed. Signal transmission in this prototype was, however, still relying on a connecting cable with the exterior of the eye, which made it unsuited for application in humans. The next step was to develop a wireless prosthesis, which Ahnad *et al*. reported in 2020 [54]. The fully diamond-encapsulated implant module was composed of a diamond electrode array (with the stimulator ASIC) and a diamond interposer (hosting a photovoltaic cell and a photodiode). The implant module was then powered and controlled using a beam of NIR laser light. 

While the results of *in vivo* tests for these devices were not yet reported, the academics developing them have partnered with a company, iBionic, for further development of the diamond-based bionic eye. A longitudinal clinical trial on patients with *retinitis pigmentosa* or choroideraemia (in a total of seven participants) is currently underway to evaluate the effectiveness of the vision resulting from the suprachoroidal implantation of the prostheses; the study will also evaluate, as a secondary outcome, the device stability and functionality with routine ophthalmic imaging, impedance, and threshold testing [55]. Market launch of this device is anticipated for 2024 [56].

## 4. Discussion 

The reports compiled in Section 2 show the utility of diamond films for cell and tissue culture. Diamond substrates with an ultrafine (2 to 10 nm) nanograin structure—UNCD—seem to be the most suitable for growing cells, which is attributed to the high density per surface area of grain boundaries, more polar than the nanodiamond grains. UNCD can support adhesion and consequent proliferation of cell types with diverse culture profiles, from fibroblasts [11,12] to NSCs. In the latter case, UNCD also exhibits bioactivity, that is, the ability to elicit a specific biological response from the cells. In this case, the NCSs started to differentiate into different lineages. Noteworthy, H-terminated UNCD spontaneously induced differentiation into neurons [6], while O-terminated UNCD favoured differentiation into oligodendrocytes [5].

NCD, having larger grain size than its UNCD counterpart and thus lower density of polar grain boundaries, can also be colonised by fibroblasts, albeit at lower cell densities. To work around this, the hydrophilicity of the surface can be increased through chemical modification of C–H endgroups (in H-terminated UNCD) to C–OH and other O-bearing endgroups (in O-terminated UNCD). This permits the growth of fibroblasts in NCD with grain size from 100 to 300 nm [12]. Similar results are reported for kidney epithelial cells MK-2 cells [18], immortalised osteoblast-like cells, and human bone marrow cells [15]. 

In addition to film morphology, it is also important to consider cell biology. Fibroblasts, for example, can easily adhere to a substrate because they contain the adequate biochemical machinery for the formation of their own ECM; immortalised cell lines are usually derived from hyperplasic cells, which have activated biochemical paths leading to facilitated cell division, increased metabolic rate, and strong survival capabilities. In turn, primary cultures of cell types that have already undergone differentiation and specialisation, such as neurons and glial cells, require specialised growth conditions involving the addition of poly-D-lysine, laminin, or other adhesion factors that promote adhesion.

The progress made on the development of diamond-based devices for retinal implantation with the aim of artificial vision is described in Section 3. While still under development, this technology is very close to reaching the market and illustrates well how biocompatibility and durability make diamond films the paramount material in future bioengineering. Regarding the use of diamond as a material for retinal implants, the crucial steps of adequate biointerfacing, biocompatibility, energy self-sufficiency, development of an adequate electronic interface, and packaging are already mastered. Results of the ongoing clinical trials will help elucidate on its efficacy, durability, and patient compliance.

## 5. Conclusions and Future Outlook

The collection of reports described in this review show how diamond films, in their morphological diversity ranging from microcrystalline diamond to NCD and even UNCD, constitute a set of materials of excellent utility in the culture of cells and tissues. The range of surface morphologies, in tandem with the possibility of fine-tuning the polarity of the surface, offer the possibility of preparing an adequate substrate for each different type of cell. Moreover, the surfaces can be treated in order to form patterns having some areas with high affinity to cells and other where the cells are less able to adhere and grow. This strategy controls and guides the growth of the cells, permitting to shape, on demand, the formation of a new tissue. 

This review shows also a most relevant example of application of diamond-based biointerface units in the construction of a functional retinal implant to restore vision. The different prototypes described in the literature all serve to illustrate the biocompatibility and excellent electric interfacing properties of diamond films, with an optimised version being currently under evaluation in human patients in a real-life scenario. One can expect to find these bionic “diamond eyes” in the market in a very near future.

## Data Availability

Not applicable.
